# Functional partitioning of sentence processing and emotional prosody in the right perisylvian cortex after perinatal stroke

**DOI:** 10.21203/rs.3.rs-3911903/v1

**Published:** 2024-02-22

**Authors:** Kelly C. Martin, Anna Seydell-Greenwald, Peter E. Turkeltaub, Catherine E. Chambers, William D. Gaillard, Elissa L. Newport

**Affiliations:** Georgetown University Medical Center, Georgetown University; Georgetown University Medical Center, Georgetown University; Georgetown University Medical Center, Georgetown University; Georgetown University Medical Center, Georgetown University; Georgetown University Medical Center, Georgetown University; Georgetown University Medical Center, Georgetown University

## Abstract

In healthy adults different language abilities—sentence processing versus emotional prosody—are supported by the left (LH) versus the right hemisphere (RH), respectively. However, after LH stroke in infancy, RH regions support both abilities with normal outcomes. We investigated how these abilities co-exist in RH regions after LH perinatal stroke by evaluating the overlap in the activation between two fMRI tasks that probed auditory sentence processing and emotional prosody processing. We compared the overlap for these two functions in the RH of individuals with perinatal stroke with the symmetry of these functions in the LH and RH of their healthy siblings. We found less activation overlap in the RH of individuals with LH perinatal stroke than would be expected if both functions retained their typical spatial layout, suggesting that their spatial segregation may be an important feature of a functioning language system.

## Introduction

Language is a uniquely human cognitive function with an unusual pattern of organization in that it is strongly lateralized to the left hemisphere (LH) in most adults^1,2^. Indeed, after a stroke to the left middle cerebral artery (MCA) in adulthood, the right hemisphere (RH) is limited in its ability to recover left-lateralized language abilities^3,4^. However, when a stroke to the left MCA causes a large cortical infarct during infancy, language abilities develop to be in the normal range^5–7^ and are lateralized to the RH^5,6,8–10^. We have administered a comprehensive battery of language assessments and functional magnetic resonance imaging (fMRI) tasks in perinatal stroke participants (henceforth LHPS participants) many years after their stroke, and they perform well in all of the language areas examined^6^: word structure, basic sentence comprehension, and comprehension of simple and complex syntax. Recently we showed in our sample of LHPS participants that auditory sentence processing engages RH regions that are precisely homotopic to typical LH language centers, and remarkably, the spatial arrangement is similar to the pattern observed in the typical dominant LH^11^.

What happens, then, to the functional map of RH perisylvian cortex if these regions have been modified to support sentence processing? A recent study in healthy young adults found that decisions about the semantic content of spoken sentences engaged LH perisylvian regions, while decisions about the emotion conveyed by the speaker’s voice (emotional prosody) engaged parallel RH regions^12^. Indeed, emotional prosody processing is often impaired after a right MCA stroke in adulthood^13^. The few studies that have examined emotional prosody after perinatal stroke have found that this ability develops to be normal, and equivalently so after a left or right MCA stroke^14,15^.

In the current fMRI investigation, we asked precisely how sentence processing and emotional prosody regions co-exist in the RH after perinatal stroke. We first investigated potential abnormalities in the diffuseness of cortical recruitment for each of these functions. For example, each function might claim a smaller amount of cortical territory than we normally observe when they lateralize to opposite hemispheres. Alternatively, one function could occupy the typical amount of space and as a consequence the other function could be confined to a smaller space than is typical. We then investigated whether sentence processing and emotional prosody occupied overlapping cortical areas in the intact hemisphere after a perinatal stroke. The fact these two functions typically lateralize to opposite hemispheres suggests that they require different properties of cortical tissue for processing their distinct functions; they may therefore need to be supported by separate subregions when only one hemisphere is available from birth.

## Results

**Figure 1:**
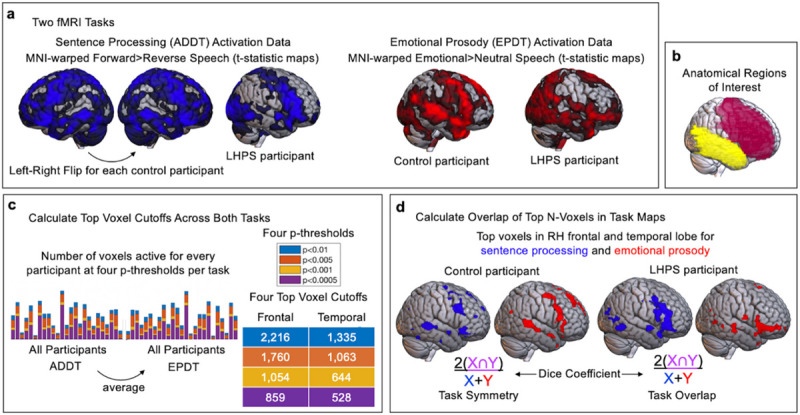
Analysis Overview. **a.** For the Auditory Description Decision Task (ADDT; left), we left-right flipped the activation map for each control participant, and left the activation map for each left hemisphere perinatal stroke participant (LHPS). For the Emotional Prosody Decision Task (EPDT; right), we did not flip the activation maps for controls or LHPS participants. **b.** We masked activation for both tasks in anatomical regions of interest and then **c.** applied a top voxel cutoff to equalize the quantity of activation within each of these regions for all participants’ activation maps for both tasks. **d.** Finally, we calculated the spatial overlap between the two activation maps (ADDT and EPDT) for each participant with a Dice Coefficient.

The present study included thirteen LHPS participants with large cortical lesions from arterial ischemic strokes to the left MCA (n = 12) or internal carotid artery (n = 1) with no additional cardiovascular disease, chronic epilepsy, or neurological disorder, and eleven healthy siblings from the same families and roughly age-matched to the LHPS participants (Table 1). Participants completed two fMRI tasks (Fig. 1a). In the Auditory Description Decision Task (ADDT)^16^ participants heard blocks of short sentences containing descriptions of common nouns (Forward Speech condition) as well as blocks of unintelligible waveforms of the same descriptions played backwards (Reverse Speech condition). In the Emotional Prosody Description Decision Task (EPDT) participants made a three-alternative forced-choice about either the emotion (happy, sad, angry; Emotional Speech condition) or sentence content (traveling, eating, or gift-giving; Neutral Speech condition). We examined activation in the temporal and frontal lobes (Fig. 1b) to cast a reasonably wide net around the perisylvian regions engaged by our tasks. All participants performed the tasks with high accuracy, with no group differences in accuracy or response time (see Methods). Subsequent steps for the ‘top voxel approach’ and analysis of spatial similarity are visualized in Fig. 1c–d and described below.

### Diffuseness of cortical recruitment for each function does not change when they must share the same hemisphere

**Figure 2:**
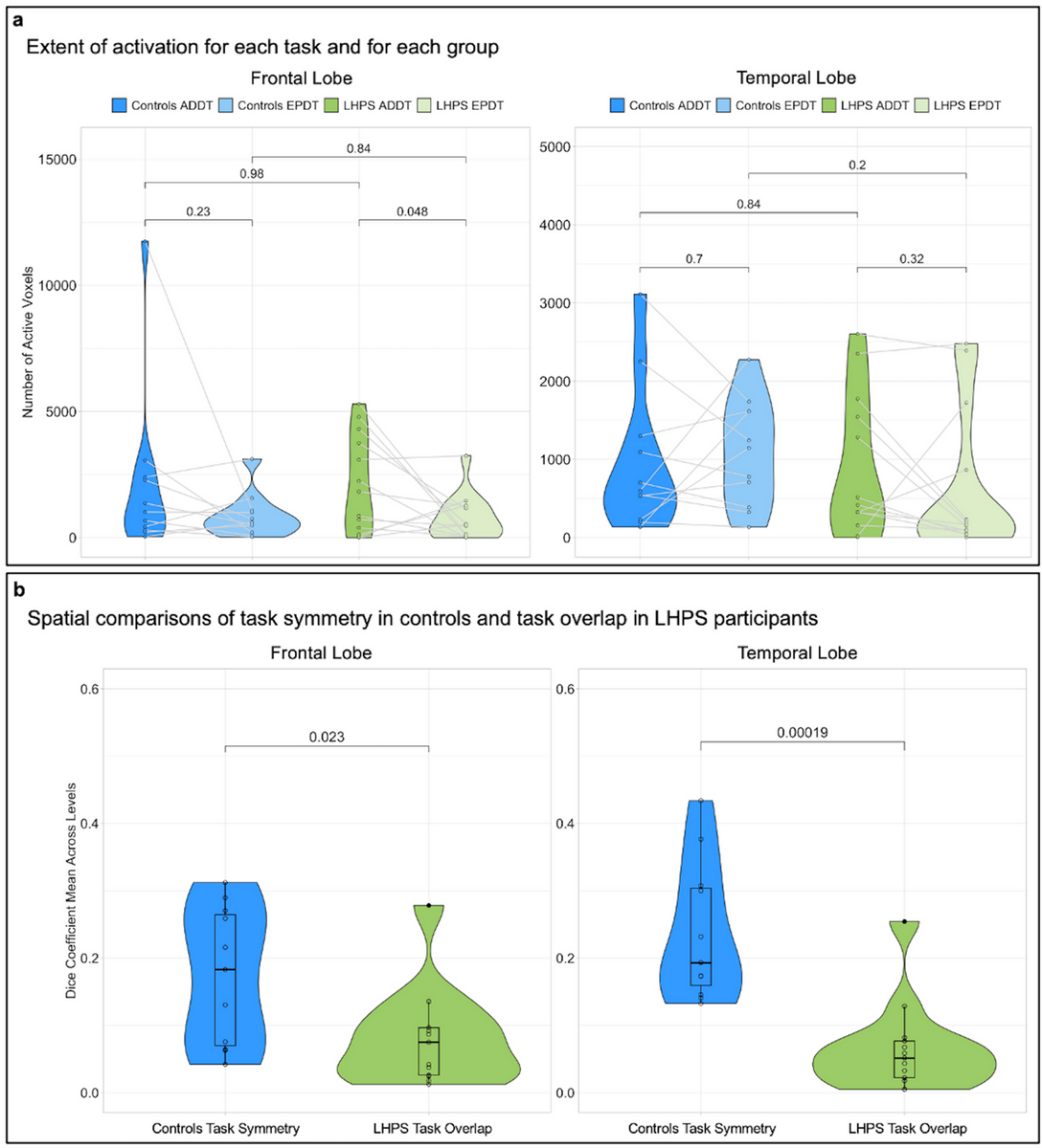
Activation diffuseness and spatial overlap. **a.** We compared the extent of activation for each task and for each group. Individual participants are labeled with open circles. Gray lines connect the data points for the different tasks for the same participant. Brackets are labeled with the p-values from two-tailed t-tests (paired within-group, independent samples between-groups). See Supplementary Fig.S2 for Spearman correlations on the number of active voxels for each task in LHPS participants and controls. **b.**The Dice Coefficient averages are shown for individual participants (circles) within each group: in blue, the symmetry of task activations in individual controls; in green, the overlap of task activations in individual LHPS participants. Brackets are labeled with the p-values from independent samples t-tests. See Supplementary Fig.S3 for the Dice Coefficient relationships at each individual top voxel level and Supplementary Fig.S4 for the Dice Coefficient relationships using a conventional thresholding approach.

First, we compared groups on the extent of activation evoked by each task (Fig. 2a). If both functions share one hemisphere by each reducing the amount of cortex they recruit, the activation for each task should be less diffuse in the preserved hemisphere of perinatal stroke participants compared to controls.

There was a significant main effect of task in the frontal lobe reflecting greater activation for sentence processing than prosody within participants (F(1, 22) = 0.008, p < 0.031), but no main effect of participant group and no interaction of participant group and task (Supplementary Table S1). Next, we examined a potential inverse relationship between the amount of activation evoked by each task in the intact hemispheres of the LHPS participants. If both functions share one hemisphere by trading off the relative amount of space they occupy, more extensive activation for one task should be linearly related to less extensive activation for the other in perinatal stroke participants but not in controls. We found no evidence of such a relationship (Supplementary Fig.S1a). These results suggest that the diffuseness of activation for sentence processing and emotional prosody is no different after perinatal stroke than it is in the healthy controls.

### Functions claim separate cortical territories when they must share the same hemisphere

We then asked, when the activation areas for the two tasks are equalized, do sentence processing and emotional prosody activations in the RH of LHPS participants overlap to the extent that would be expected if the typical LH activation for sentence processing were simply mirrored into the RH? We employed a ‘top voxel’ approach (Fig. 1c)^17,18^ which allows us to compare the same number of the most active voxels between participants and between hemispheres to evaluate similarity in the spatial arrangements of activity (measured using a Dice Coefficient; Fig. 1d). Importantly, this approach enforces that the areas we compare will be identical in size: this means that the voxels may differ in their absolute activation (as is common between different tasks and different participants) but if the most consistently active voxels (those with the highest t-values) for each task fall in the same spatial area, we can quantify that both tasks have a similar spatial distribution regardless of idiosyncrasies in activation magnitude.

**Figure 3:**
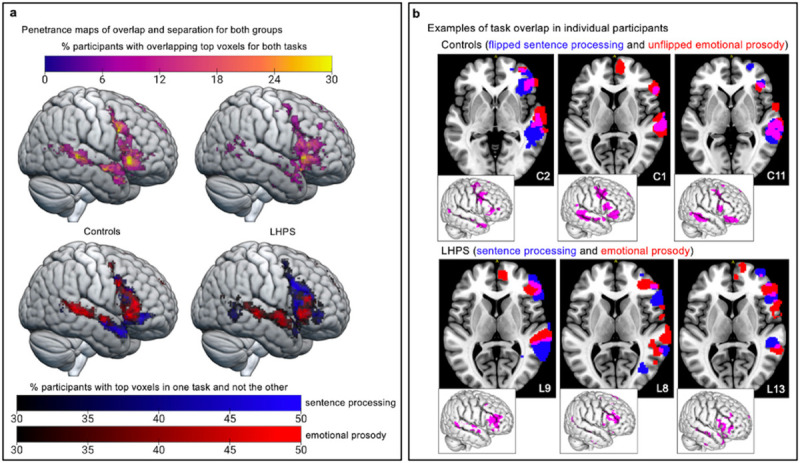
Individual participant maps and group penetrance maps of activation overlap and separation. **/ a.**In the task overlap penetrance maps (top row), yellow areas reflect regions with greater consistency (a greater number of participants) in task overlap or symmetry across the group. In the task separation maps (bottom row), brighter blue areas reflect greater consistency for sentence-processing-only top voxels, while brighter red areas reflect greater consistency for prosody-only top voxels. Brain areas exhibiting consistent overlap or separation are summarized in Supplementary Table S2. **b.** Axial slices and sagittal renderings of the top voxel maps for each task and their overlap are overlaid onto the MNI template for three control participants (top row) and three LHPS participants (bottom row). There was significantly less overlap between tasks in the RH of LHPS participants than was measured in controls (quantified in Fig.2).

Task symmetry in controls was evaluated by comparing their RH prosody activations to their left-right flipped sentence processing activations, which serves as a proxy for how overlapping these systems could theoretically be if, after a LH perinatal stroke, typically left-lateralized language functions were simply transposed into the RH. Task overlap in LHPS participants was lower than task symmetry in controls in both the right frontal (t(17.7) = 2.49, p = 0.0229) and temporal lobes (t(16.6) = 4.77, p = 0.0002; Fig. 2b). These Dice Coefficients were measured at four top voxel levels and the average across levels was compared between participants (however, the same relationships persist at each level and using a conventional thresholding approach (Supplementary Fig.S2&S3). Examples of the unique spatial layouts for these two tasks in LHPS participants illustrate that sentence processing and emotional prosody activate nearby but minimally overlapping areas, as compared with the task symmetry in the example controls (Fig. 3b). Group maps of task separation (Fig. 3a) reveal that in many LHPS participants emotional prosody claimed much of the mid-to-posterior superior temporal gyrus, while sentence processing claimed part of the posterior middle temporal gyrus (regions of task overlap and separation listed in Supplementary Table S2).

## Discussion

In summary, we found that sentence processing and emotional prosody claim separate cortical territories in RH perisylvian cortex after a LH perinatal stroke, rather than developing a sentence processing system that is entirely superimposed onto emotional prosody regions. Based on findings in this population from our group and others, it is evident that the young brain after a perinatal stroke is capable of developing a fully functioning language system in right perisylvian cortex, one that is much like that of a typical LH. The RH organization of language processes after LH perinatal stroke may leverage language-processing potential that RH regions are typically equipped with early in life^5,19,20^.

As a caveat, our Emotional > Neutral Speech contrast eliminates from attention those areas that are active during both emotional prosody and sentence processing. While there are undoubtedly also areas that are active during both tasks, the areas that *preferentially* process each type of information consistently segregate in the RH of LHPS participants. It is unknown whether this outcome involves a change to the typical layout of perisylvian cortex that may exist at the earliest stages of brain and cognitive development. We also cannot determine the presence or absence of behavioral crowding for these two cognitive abilities because the present study focused on the cortical mapping of these abilities using simple tasks that did not probe the boundaries of performance.

Our findings have important implications for our understanding of how cognitive functions become mapped to their neural substrates during development and after cortical injury. Specialized systems for sentence processing and emotional prosody, either in separate hemispheres or in subregions of the sole intact perisylvian cortex, may emerge through competitive specialization^21,22^. In one instantiation of this computational framework^23^, whichever system initially processes a given dimension of the input (e.g., lexicosemantic features) more accurately will undergo slight changes that increase the likelihood that it will process the next instance of that same dimension when a task demands it. Simultaneously, the other system undergoes changes that decrease the likelihood that it will process the next instance of that same dimension, and increase the likelihood that it will process a different dimension of the input (e.g., emotional prosody). This type of bias-strengthening through repeated exposure may also explain how the left-lateralization of language processing becomes more inflexible with age.

## Methods

### Participants

The data for the current study were collected as part of the Pediatric Stroke Research Project at Georgetown University^5,6^. The study was approved by the Institutional Review Board at Georgetown University Medical Center. All research was performed in accordance with relevant guidelines/regulations; all participants provided informed consent (adults) or parental informed consent and child assent (children). For the present analyses, thirteen perinatal arterial ischemic stroke participants (six female) were included, all of whom suffered a large cortical stroke to the left middle cerebral artery (the left internal carotid for one participant) with no additional diagnosis of cardiovascular disease, chronic epilepsy, or neurological disorder other than perinatal stroke. Eleven healthy controls (three female) were also included, who are siblings from the same families and roughly age-matched to the stroke participants. We did not include three additional participants that were included in prior analyses^6^: one stroke participant and one healthy control were too young (we applied an age cutoff of 10 years old for the current study to ensure that sentence processing was fully lateralized^24^, and one stroke participant’s prosody scan data was unavailable at the time of this analysis. See Newport et al., 2022 for more detailed information about participant inclusion and language outcomes.

Magnetic Resonance Imaging

## Auditory Description Decision Task (ADDT)

The Auditory Description Decision Task (ADDT) is a sentence processing task developed by Gaillard and colleagues^16,24,25^ and was modified for use in the Pediatric Stroke Research Project^5,6^. Briefly, participants heard blocks of short sentences containing auditory descriptions of common nouns (e.g., A large gray animal is an elephant) and were instructed to press a button if the description was correct (Forward Speech condition). They also heard blocks of unintelligible waveforms of the same descriptions played backwards and were instructed to press a button when a tone followed the auditory sequence (Reverse Speech condition). Each run (5:48 in duration) contained alternating blocks of Forward and Reverse Speech conditions (30 seconds each), beginning with Reverse Speech, with 12-second silent periods interspersed between blocks. Button presses were prompted on 50% of Forward Speech stimuli and 50% of Reverse Speech stimuli. Stimuli in each block were presented every 5 seconds, with a 3-second stimulation period followed by a 2-second response window. Runs were re-collected if motion was excessive, and two runs were analyzed for each participant. For the Forward Speech condition, controls had a mean accuracy of 97.5% +/−2.4% (93.8–100.0%) (reported as mean +/−standard deviation, in parentheses range) and LHPS participants had a mean accuracy of 95.2% +/−6.1% (79.2–100.0%). For the Reverse speech condition, controls had a mean accuracy of 99.2% +/−1.4% (95.8–100.0%) and LHPS participants had a mean accuracy of 97.9% +/−2.1% (93.8–100.0%). Independent samples t-tests did not reveal differences between participant groups in the response times for the Forward or Reverse conditions.

## Emotional Prosody Decision Task (EPDT)

The emotional prosody decision task (EPDT) was designed by Seydell-Greenwald and colleagues^12^ to be similar in structure to the ADDT but with alternating Emotional Speech and Neutral Speech conditions. During the Emotional Speech condition, participants heard short sentences spoken in one of three emotions (happy, sad, or angry) which were each followed by one of three visual cues (sun, raindrop, or boxing glove). During the Neutral Speech condition, participants heard short sentences whose semantic content was about one of three activities (traveling, eating, or gift giving) spoken in a neutral tone. These sentences were followed by a visual cue representing one of the three activities (car, plate and utensils, gift box). Participants were instructed to press a button when the visual cue matched the speaker’s emotion (50% of Emotional Speech sentences) or the speaker’s activity (50% of Neutral Speech sentences). All participants completed two runs of the task. Each run (5:00 duration) contained eight 24-second blocks beginning with three seconds of written instructions and comprised of 6 sentences (each roughly 2 seconds in duration, followed by a 2 second response period). Silent periods (12 seconds long) were interspersed between blocks. Runs were repeated if motion was excessive, and two runs were analyzed for each participant. For the Emotional Speech condition, controls had a mean accuracy of 92.6% +/−6.7% (81.3–100.0%) and LHPS participants had a mean of 92.3% +/−7.0% (77.1–100.0%). For the Neutral Speech condition, controls had a mean of 96.8% +/−2.5% (91.7–100.0%) and LHPS participants had a mean of 91.3% +/−6.7% (79.2–100.0%). Independent samples t-tests did not reveal differences between participant groups in the response times for the Emotional or Neutral conditions.

## Scanner and Auditory Equipment

Participants were scanned on a 3 Tesla Siemens MAGNETOM Trio scanner using a 12-channel headcoil at Georgetown University’s Center for Functional and Molecular Imaging. Three participants were scanned after a scanner upgrade to a Prisma model with a 20-channel head coil. Auditory stimuli for both tasks were delivered through Sensimetrics Model S14 insert headphones. Participants were also fitted with additional Bilsom ear defenders to reduce scanner noise, and they confirmed that they could clearly hear the task stimuli.

## Scan Sequences

A high-resolution anatomical image was collected and was repeated if motion was excessive: Siemens MPRAGE, 176 sagittal slices, TR = 2.53s, TE = 3.5ms, flip angle = 7 deg, 1×1×1mm voxels, whole brain coverage. While participants completed the ADDT, 116 whole brain volumes were collected for each of the two functional runs: functional echo-planar images, 50 horizontal slices, descending order, TR = 3s, TE = brain, flip angle = 90 deg, 3×3×3mm voxels. One of the control participants completed an earlier version of the task, which included 120 whole brain volumes. The 100 whole brain volumes collected for each of the two runs of the EPDT had the same acquisition parameters as the ADDT.

## fMRI Processing

The MRI scans in the current study were processed using SPM-12 (Wellcome Trust Centre for Neuroimaging at University College London, https://www.fil.ion.ucl.ac.uk/spm/doc/). Preprocessing included slice-timing correction, realignment to the middle volume of each run, co-registration to the native-space anatomical image, spatial normalization to the MNI-152 average template (resulting resolution 2mm), and then smoothing (8mm FWHM Gaussian kernel). The fMRI time-courses for voxels inside the brain were statistically modeled using a general linear model that included the two conditions of interest (Forward and Reverse Speech, or Emotional and Neutral Speech) which were convolved with a canonical hemodynamic response function, as well as motion estimates for rotation and translation along the x-, y-, and z-axes, and a high-pass filter for the duration of the task run. For each task, we then calculated voxel-wise t-tests on the beta maps for the conditions of interest to generate statistical contrast maps for Forward > Reverse Speech (sentence processing) activity and Emotional > Neutral Speech (emotional prosody) activity (Fig. 1a in the main text).

## Flipped ADDT Activation Maps for Controls

Part of our current investigation involved quantifying the degree of symmetry or expected overlap between the LH activation for sentence processing and the RH activation for emotional prosody processing in the healthy brain. To calculate this symmetry or expected overlap, we flipped each control’s structural and functional images across the midline (using SPM-12’s reorient utility) before preprocessing (Fig. 1a in the main text). Their flipped LH activity was then co-registered to their flipped LH anatomy. Then, in order to address minor structural asymmetries between the two hemispheres^26^, we spatially normalized the flipped images to the MNI template anatomy such that the participant’s flipped LH activation was warped to the template’s RH anatomy.

## Regions of Interest (ROIs)

The frontal and temporal lobe anatomical masks from the WFU-Pick Atlas^27^ were resliced to match the dimensions of the MNI-space anatomical and statistical maps and were used to isolate frontal and temporal activations (Fig. 1b in the main text).

## Extent of Activation

We first asked whether the activation was more or less diffuse for sentence processing or emotional prosody processing when both functions were lateralized to the same hemisphere compared to their typical bi-hemispheric distribution (Fig. 2a in the main text). We thresholded every participant’s statistical map for each task (voxelwise p < 0.001 with minimal clustering, k≥4) and counted the number of active voxels in the frontal and temporal ROIs. For controls, we counted the active voxels in the LH for sentence processing and in the RH for emotional prosody processing. For LHPS participants, we counted the active voxels for each task in the RH. By comparing the number of active voxels in controls to LHPS participants for each task, we can determine whether the diffuseness of sentence processing or emotional prosody activation may be different after a perinatal stroke. We also examined the relationship in the amount of activation for both tasks among LHPS participants to determine whether there was an inverse relationship. If so, it would suggest that across LHPS participants, one function activated a smaller area when the other activated a larger area within RH perisylvian cortex.

## Top Voxel Approach

We also investigated the amount of spatial overlap in the activation for these two tasks when we compared the same number of the most active voxels for both tasks across all participants. As we have described in previous work^17^ (and KM, AS-G, PT, WG, EN, et al. (2023) in press), equalizing the number of voxels being compared between participants and between tasks simplifies the interpretation of activation overlap/non-overlap. Briefly, the goal is to obtain one value for each person that quantifies the amount of overlap between the two task maps. When the two maps are the same size for every person, the amount of overlap for each person is a straightforward percentage that reflects the size of the intersecting area for both maps and is directly comparable between people.

First, we applied four different statistical thresholds (p < 0.01, p < 0.005, 0 < 0.001, p < 0.0005) with minimal clustering (k≥4) to every participant’s ADDT (Forward > Reverse Speech) and EPDT (Emotional > Neutral Speech) statistical map. Within each ROI, we found the number of voxels that survived at each threshold for each task. For controls, we counted the active voxels in the LH for the ADDT and in the RH for the EPDT. In LHPS participants, we counted the active voxels for each task in the RH. We averaged this voxel count for both tasks across all participants at each of the four thresholds, which produced four top voxel cutoffs (Fig. 1c in the main text). For each participant, we then ranked the t-values in the ROI from highest to lowest and generated a new constrained map that included the top N voxels in each ROI for each task. We repeated this for each top voxel cutoff. As a result of this procedure, for each of the four cutoffs, every participant had a set of size-matched ADDT and EPDT maps in the frontal and temporal lobe (Fig. 1d in the main text). We also analyzed the spatial overlap using a conventional thresholding approach at four p-thresholds and included the results in the Supplementary Materials for comparison.

## Spatial Overlap Analysis (Dice Coefficient)

We measured spatial overlap between the two tasks’ activation maps by calculating a Dice Coefficient for each participant, which is a ratio of the number of overlapping voxels for the two activation maps (x and y) relative to the total number of active voxels in both maps (Fig. 1d in the main text):

$$\frac{2\left(x\cap y\right)}{x+y}$$

We calculated a Dice Coefficient for each ROI separately at each top voxel cutoff. We then averaged these Dice Coefficients calculated across all four cutoffs to obtain one summary value of spatial overlap within each region for each participant that was reasonably unbiased. For control participants, we measured the spatial overlap between their flipped LH ADDT map and their unflipped RH EPDT map to quantify how symmetrical the task activations were in each person. For LHPS participants, we measured the spatial overlap between their RH ADDT and EPDT maps to quantify how overlapping the activation areas were in each person’s intact hemisphere (Fig. 2b in the main text).

## Penetrance Maps

Penetrance maps (Fig. 3 in the main text) visualize where spatial overlap was most consistent across participants in each group. We created task overlap penetrance maps and task separation penetrance maps, which show the brain areas that were most commonly active for both tasks or most commonly active for one task but not the other, respectively. At each voxel of the task overlap penetrance maps, there is a percentage value reflecting the number of participants who had task overlap at that voxel. At each voxel of the task separation maps, there is a percentage value reflecting the number of participants who had activation for one task but not the other (activation meaning it was one of the top voxels for one task but not the other) at that voxel. These maps were rendered on the MNI-152 standard template using MRIcroGL (https://www.nitrc.org/projects/mricrogl).

## Statistical Comparisons

Statistical analyses were performed using R through R-Studio (http://www.R-project.org/). For our analysis of activation extent, we performed two types of statistical tests. First, we calculated two-way repeated measures ANOVAs to measure the effect of task (ADDT or EPDT, within-subject) and group (LHPS or Control, between-subjects) as well as the interaction between task and group on the amount of activation measured (Fig. 1a in the main text). Second, we calculated Spearman correlations on the extent of activation for the ADDT and the EPDT across LHPS participants to determine whether a larger area recruited by sentence processing correlated with a smaller area recruited by emotional prosody processing (or the reverse). We also performed Spearman correlations on the extent of activation in each hemisphere for control participants. These correlations are included in Extended Data Fig. 1. For our analysis of spatial overlap, using the top voxel-constrained maps, we calculated two-tailed independent samples t-tests in the frontal and temporal ROIs to compare the Dice Coefficients measuring task overlap in LHPS participants to the Dice Coefficients measuring task symmetry in controls (Fig. 2b in the main text). This allowed us to evaluate whether the overlap for these tasks in the RH of LHPS participants was less than would be expected if sentence processing was physically transposed into the RH in the healthy brain.

## Figures and Tables

**Table 1:** Participant characteristics

	n	Age (years)	Handedness	L1	Sex	Stroke Area
LHPS	13	10.0–25.3, mean: 17.22, sd: 4.39	13 L	English	6 F	12 middle cerebral artery, 1 internal carotid
Controls	11	11.5–29.5, mean: 15.95, sd: 5.0	1 L	English	3 F	N/A

## Data Availability

The datasets used and/or analysed during the current study are available from the corresponding author on reasonable request.
